# Error Compensation Method for Pedestrian Navigation System Based on Low-Cost Inertial Sensor Array

**DOI:** 10.3390/s24072234

**Published:** 2024-03-30

**Authors:** Lijia Cao, Xiao Luo, Lei Liu, Guoqing Wang, Jie Zhou

**Affiliations:** 1 School of Automation & Information Engineering, Sichuan University of Science & Engineering, Zigong 643000, China; 321085404420@stu.suse.edu.cn (X.L.); 322085404507@stu.suse.edu.cn (L.L.); 323085404223@stu.suse.edu.cn (G.W.); 323085404431@stu.suse.edu.cn (J.Z.); 2 Key Laboratory of Higher Education of Sichuan Province for Enterprise Informationalization and Internet of Things, Zigong 643000, China; 3 Artificial Intelligence Key Laboratory of Sichuan Province, Zigong 643000, China

**Keywords:** IMU array, IMU calibration, error compensation, pedestrian navigation

## Abstract

In the pedestrian navigation system, researchers have reduced measurement errors and improved system navigation performance by fusing measurements from multiple low-cost inertial measurement unit (IMU) arrays. Unfortunately, the current data fusion methods for inertial sensor arrays ignore the system error compensation of individual IMUs and the correction of position information in the zero-velocity interval. Therefore, these methods cannot effectively reduce errors and improve accuracy. An error compensation method for pedestrian navigation systems based on a low-cost array of IMUs is proposed in this paper. The calibration method for multiple location-free IMUs is improved by using a sliding variance detector to segment the angular velocity magnitude into stationary and motion intervals, and each IMU is calibrated independently. Compensation is then applied to the velocity residuals in the zero-velocity interval after zero-velocity update (ZUPT). The experimental results show a significant improvement in the average noise performance of the calibrated IMU array, with a 3.01-fold increase in static noise performance. In the closed-loop walking experiment, the average horizontal position error of a single calibrated IMU is reduced by 27.52% compared to the uncalibrated IMU, while the calibrated IMU array shows a 2.98-fold reduction in average horizontal position error compared to a single calibrated IMU. After compensating for residual velocity, the average horizontal position error of a single IMU is reduced by 0.73 m, while that of the IMU array is reduced by 64.52%.

## 1. Introduction

With the rapid advancement of technology, pedestrian navigation systems have attracted considerable attention from researchers due to their wide range of applications in both military and civilian contexts [1,2]. These systems can be classified into two categories based on their reliance on external signals: those that rely on external signals and those that do not [3,4]. The pedestrian navigation system that relies on external signals uses signals from Global Navigation Satellite Systems (GNSSs), Bluetooth, and others to determine the pedestrian’s location and provide navigation guidance [5,6,7,8,9]. However, in scenarios such as fires or earthquakes, where external signals are obstructed, these systems fail to provide accurate navigational positioning, and the installation cost of communication equipment is typically high [10,11,12]. On the other hand, systems that do not rely on external signals primarily use the inertial measurement unit (IMU) of micro-electromechanical systems (MEMSs) to measure relevant information about pedestrian movement for positioning [13]. Due to their independence from external signals, inertial navigation systems exhibit higher reliability and stability, even in harsh environments [14,15].

However, consumer-grade MEMSs suffer from design and manufacturing flaws that lead to significant measurement errors in their output values. These errors accumulate over time during pedestrian navigation, severely affecting the accuracy of the system [16]. Correcting and compensating for these errors is necessary to achieve higher accuracy in pedestrian navigation. Methods for correcting and compensating for IMU measurement errors include calibrating the IMU and then fusing the measurements from multiple IMUs to form an IMU array [17]. According to the theory of random error, fusing data from N IMUs can reduce the random error by a factor of $1/\sqrt{N}$ compared to a single IMU and achieve higher measurement accuracy [18]. Therefore, constructing an IMU array by combining multiple IMUs can significantly improve measurement accuracy, while the redundancy of MEMS further enhances measurement reliability.

Researchers have shown great interest in the potential of IMU arrays to suppress noise and improve the accuracy of pedestrian navigation. Skog et al. designed an array comprising 18 IMUs and discussed its potential benefits. They also used Allan variance analysis to evaluate the error characteristics of the array when in a stationary state [19]. In addition, various arrays have been designed by researchers to quantitatively analyze static noise performance [20,21]. The results indicated a significant improvement in the static noise performance of the arrays. However, these studies focused primarily on static noise performance and did not address dynamic errors, such as scale factor and cross-axis coupling. Blocher et al. conducted navigation experiments using an array of 14 IMUs to address this issue. They were able to improve navigation accuracy by calibrating the scale factor, offset, and cross-axis sensitivity of the IMU devices [22]. However, the improvement in dynamic performance was less than expected. To further improve navigation accuracy, Wang et al. used an array of 16 IMUs for integrated navigation and achieved a 3.4-fold increase in accuracy through precise calibration compensation using a turntable [23]. However, relying solely on a turntable for calibration has limitations in terms of cost and accuracy. Hussein et al. proposed an on-site manual calibration method for IMU arrays and used a specialized Kalman filter to estimate error parameters to overcome these issues. They evaluated the performance of the calibrated array for integrated navigation [24]. However, the applicability of the error model of this method is limited and not widely applicable. On the other hand, Carlsson et al. introduced an IMU array calibration compensation method based on maximum likelihood estimation, which significantly improved the motion estimation accuracy [25]. However, maximum likelihood estimation typically requires a large amount of data for parameter estimation, which may take considerable time to collect. In conclusion, despite some studies attempting compensation for IMU arrays, challenges remain, including accurate compensation for dynamic errors and problems related to the cost and applicability of calibration methods.

The simplest method of fusing data in an IMU array within the data fusion algorithm is to calculate the average value of the raw IMU measurements. Researchers have proposed various data fusion methods for IMU arrays and simple averaging fusion. Skog et al. presented an IMU array data fusion method based on maximum likelihood estimation [26]. At the same time, Shen et al. introduced an optimal bounded ellipse (OBE) algorithm using a relaxed Chebyshev center (RCC) to fuse signals from gyroscope arrays [27]. In addition, Dusan et al. proposed an adaptive sensor weighting adjustment method based on the root mean square error (RMSE) of the weighted average value of all sensors. This method suppresses degraded sensors while maintaining the overall estimation accuracy [28]. Li et al. also developed a comprehensive framework that combines adaptive dead reckoning (ADR) with zero-velocity update (ZUPT). This framework selects and eliminates IMUs with significant drift errors based on the array step length and heading angle calculated by each IMU [29]. Although these methods have demonstrated their effectiveness in data fusion of IMU arrays, further validation of their performance in dynamic experiments is required as their effectiveness may not meet expectations. Despite the availability of several options for data fusion algorithms in IMU arrays, there is still room for improvement in their practical effectiveness in dynamic navigation.

Many previous studies have overlooked the compensation and suppression of IMU systematic errors [19], which significantly affect the measurement accuracy of IMUs. Even when the measurement data from each IMU is integrated, the effects of these errors remain unmitigated, severely compromising the accuracy of pedestrian navigation. Therefore, despite the potential of IMU arrays to improve static noise performance, the measurement accuracy of IMU arrays falls short of expectations due to the lack of calibration compensation for systematic errors within each IMU.

Systematic error compensation primarily falls into two main categories. First, for an estimation of system errors by Kalman filtering, Gao et al. proposed various stochastic weighting methods to estimate system errors in the observation model of dynamic vehicle navigation. They compensated for system model errors by correcting observation residual vectors and state noise vectors during the filtering process [30,31,32]. In addition, Gao et al. introduced a novel transfer alignment robust adaptive filtering method that adaptively adjusts and updates prior information through equivalent weighting matrices and adaptive factors to counteract the influence of system model errors on system state estimation, thereby improving the accuracy of state parameter estimation [33]. Second, it compensates and suppresses systematic errors through IMU calibration. In this paper, calibration is performed on IMUs to compensate for systematic errors within them.

**Figure 1:**
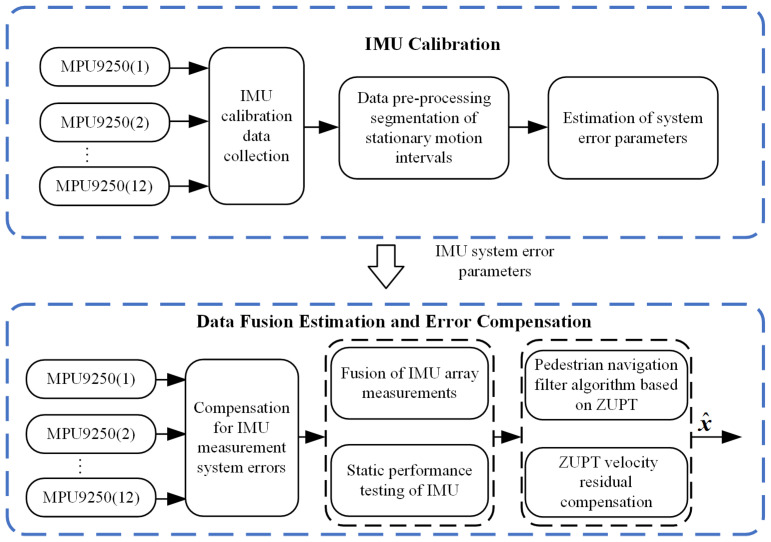
Compensation Framework for Pedestrian Navigation System Based on IMU Array.

This paper aims to improve the navigation performance of the system by calibrating and compensating for the velocity residual of a custom-designed IMU array. Traditional multi-position IMU calibration methods have not paid much attention to the accurate segmentation of stationary and moving intervals. However, the accuracy of segmentation directly affects the calibration accuracy of IMUs. To address this, we designed a sliding variance detector for angular velocity magnitude to improve the segmentation accuracy and hence the calibration accuracy of IMUs. In addition, velocity residual position errors after ZUPT, which are often overlooked in pedestrian navigation research, can accumulate over time. We treat the displacement in zero speed intervals as pseudo measurements and compensate for position errors caused by velocity residual. The general framework of this process is illustrated in Figure 1. First, each IMU in the array undergoes individual calibration to estimate systematic error parameters, thereby compensating for the systematic errors of the IMU array. Second, the measurements from the IMU array are fused, and the static noise performance is evaluated. In addition, a ZUPT algorithm is applied to correct pedestrian navigation and to compensate for position errors due to residual velocity after ZUPT. Finally, the pedestrian navigation performance is evaluated. The proposed method improves IMU array data fusion and pedestrian navigation accuracy by integrating IMU calibration and velocity residual compensation. This advancement enables the IMU array to produce more accurate measurement results, thereby improving the overall accuracy performance of pedestrian navigation. Consequently, this research is crucial for the implementation of IMU arrays in pedestrian navigation and provides valuable references for future related studies.

## 2. IMU Array Design

The shape, size, and number of MEMSs are important factors to consider when designing an IMU array. Different sizes and shapes of arrays require different numbers of MEMSs. If the MEMSs are distributed on a two-dimensional plane, at least 3 three-axis accelerometers are required to extract the rotation information of the three axes of the IMU array. Too many MEMSs will increase power consumption and cost, while the synchronization of data acquisition in the IMU array is not guaranteed. Therefore, after considering various factors, a balance must be struck between the reliability of data collection and the power consumption cost of the IMU array.

**Figure 2:**
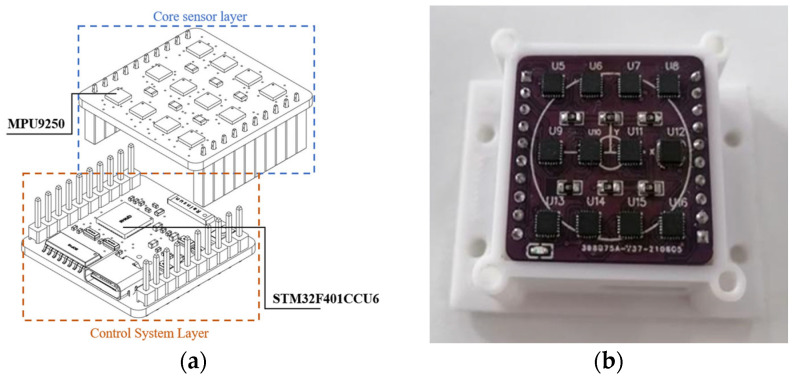
IMU array system design: (**a**) IMU design diagram and (**b**) IMU physical picture.

As shown in Figure 2, the IMU array consists of two layers that help reduce the footprint of the IMU array, improve portability, and better adapt to pedestrian navigation applications. The upper layer, which is the core sensor layer, consists of 12 MPU9250 sensors. The MPU9250 sensor is manufactured by TDK InvenSense, located in California, United States. Each MPU9250 sensor can transmit data using the SPI or I2C protocols. Since the system needs to read data from 12 MPU9250 sensors simultaneously, using the SPI protocol to read data simultaneously would consume many GPIO resources. Therefore, the I2C protocol is used for data transfer, using one clock line (SCL) and 12 data lines (SDA), saving GPIO resources and increasing the sampling frequency, resulting in higher synchronization performance. The lower layer comprises the control system layer, consisting of the STM32F401CCU6 processor and its auxiliary circuitry. The power circuit is provided by the RT9013-33GB chip, a low dropout (LDO) regulator with a fixed 3.3-V output suitable for common dropout applications in power management systems. Packaged in the SOT23-5 package, the device is ideal for limited board space applications. It has good current capability and heat dissipation, making it suitable for low power management in portable devices and consumer electronics.

Due to the lack of temperature error compensation in this paper, the theoretical noise performance of the custom-developed IMU array is compared to the commercially available MTi-2 and ADIS16500 IMUs. The MTi-2 sensor is manufactured by Xsens, a subsidiary of Movella based in the United States. The ADIS16500 sensor is manufactured by Analog Devices, Inc. located in Massachusetts, United States. Based on the performance parameters provided by the manufacturers, comparisons are made in terms of the noise performance of the accelerometers and gyroscopes, as well as the price of the IMUs. The comparison results at 25 °C are shown in Table 1 and indicate that the custom IMU array reduces the cost by 64.60% and 65.22% compared to the other two IMUs. The theoretical noise density of the custom IMU array for gyroscopes is lower than the other two IMUs and close to that of the MTi-2, with a reduction of 52.46% compared to the ADIS16500. The accelerometer noise density is similar to the other two IMUs. Based on the comparison, it can be concluded that the low-cost array designed in this paper significantly reduces the cost compared to the mainstream IMU modules on the market, while its performance is also comparable to the compared IMU modules.

## 3. IMU Array Calibration

Measurement error in MEMSs consists of two main components: random error and systematic error. Random errors include sources such as noise and drift, which introduce potential inaccuracies in the measurement results. Reference [34] suggests that by fusing the measurements from an IMU array, it is possible to reduce and compensate for the random errors of the IMUs. However, the systematic errors of individual IMUs cannot be calibrated or compensated for. Systematic errors are inherent flaws in the measurement process that produce consistent biases across successive measurements rather than being randomly distributed. Examples of MEMS systematic errors include bias, scale factor, and non-linearity errors. As mentioned in reference [35], these errors can be compensated for by IMU calibration.

The multi-position, device-free IMU calibration method described uses known motion conditions and attitude changes to calibrate the IMU. This approach eliminates the need for expensive equipment such as turntables, resulting in cost savings. It also allows real-time calibration in specific environments, such as outdoors, minimizing errors caused by environmental factors [36]. Local gravity can be determined by the gravitational force experienced by a stationary accelerometer. The measured acceleration value from the accelerometer includes gravity, system errors (such as bias and scale factor), and noise. The static interval is minimized. Once the accelerometer is calibrated, the gravity vector it measures is used as a reference to calibrate the gyroscope. By integrating the angular velocity between two successive static intervals, the rotated gravity direction can be estimated, and the estimated gravity direction is adjusted based on the reference error provided by the accelerometer. This process allows the error parameters of the gyroscope to be obtained. The key to achieving accurate multi-position, device-free calibration is to distinguish between the static and dynamic intervals.

### 3.1. IMU Error Model

**Figure 3:**
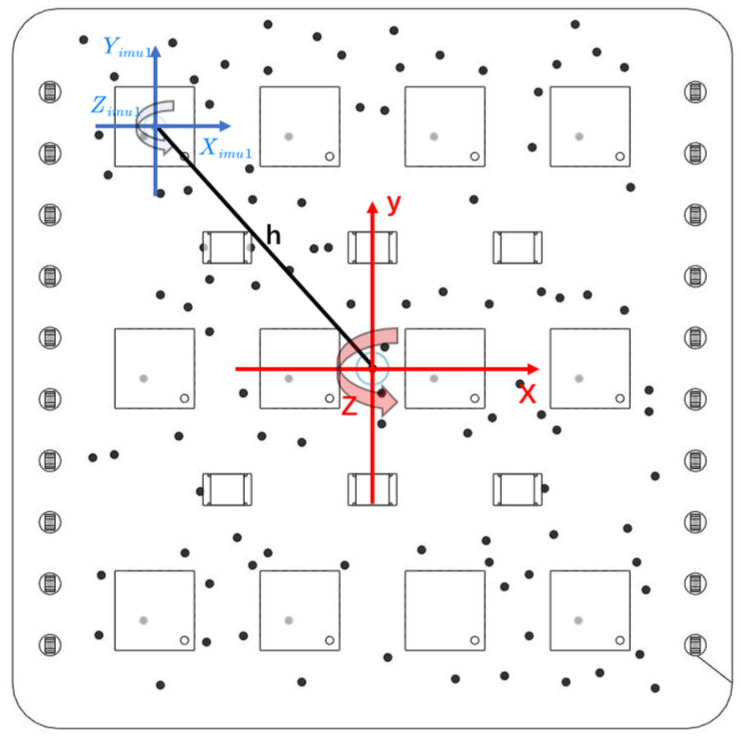
IMU array coordinate system.

To calibrate an IMU, it is necessary to first establish an error model for the IMU. As shown in Figure 3, the coordinate system for each IMU is denoted as $b_{i}$, with the center point of each chip serving as its coordinate origin. Here i=1,2⋯12, and an array coordinate system B is established with the center of the IMU array. Due to installation errors during soldering, non-orthogonal angular errors exist between the $b_{i}$ coordinate system of each IMU and the array coordinate system B [37]:

(1) $$s^{B}=Ts^{bi},T=\left[\begin{array}{ccc} 1 & -\beta_{yz} & \beta_{zy}\\ \beta_{xz} & 1 & -\beta_{zx}\\ -\beta_{xy} & \beta_{yx} & 1 \end{array}\right]$$

here $s^{B}$ and $s^{bi}$ represent the IMU array coordinate system and the coordinate system of each IMU to specific force or angular velocity, respectively, and $\beta_{ij}$ is the non-orthogonal angle.

Assuming that the coordinate system of the IMU array coincides with the orthogonal coordinate system of the accelerometer, the non-orthogonal error matrix of the accelerometer can be obtained by transforming Equation (1):

(2) $$f^{B}=T^{f}f^{bi},T^{f}=\left[\begin{array}{ccc} 1 & -\alpha_{yz} & \alpha_{zy}\\ 0 & 1 & -\alpha_{zx}\\ 0 & 0 & 1 \end{array}\right]$$

where $f^{B}$ and $f^{b_{i}}$ represent the specific force in coordinated systems B and $b_{i}$.

For the gyroscope, its non-orthogonal error matrix is given by:

(3) $$\omega^{B}=T^{g}\omega^{bi},T^{g}=\left[\begin{array}{ccc} 1 & -\gamma_{yz} & \gamma_{zy}\\ \gamma_{xz} & 1 & -\gamma_{zx}\\ -\gamma_{xy} & \gamma_{yx} & 1 \end{array}\right]$$

where $\omega^{B}$ and $\omega^{b_{i}}$ represent the angular velocity in coordinated systems B and $b_{i}$.

The scale factor errors for the accelerometer and gyro can be respectively defined as:

(4) $$K^{f}=\left[\begin{array}{ccc} s_{x}^{f} & 0 & 0\\ 0 & s_{y}^{f} & 0\\ 0 & 0 & s_{z}^{f} \end{array}\right],K^{g}=\left[\begin{array}{ccc} s_{x}^{g} & 0 & 0\\ 0 & s_{y}^{g} & 0\\ 0 & 0 & s_{z}^{g} \end{array}\right]$$

The zero bias for the accelerometer and gyro can be respectively represented as follows:

(5) $$b^{f}=\left[\begin{array}{c} b_{x}^{f}\\ b_{y}^{f}\\ b_{z}^{f} \end{array}\right],b^{g}=\left[\begin{array}{c} b_{x}^{g}\\ b_{y}^{g}\\ b_{z}^{g} \end{array}\right]$$

The complete error calibration model for IMU is as follows:

(6) $$f^{B}=T^{f}K^{f}\left(f^{bi}+b^{f}+\upsilon^{f}\right)$$

(7) $$\omega^{B}=T^{g}K^{g}\left(\omega^{bi}+b^{g}+\upsilon^{g}\right)$$

here $\upsilon^{f}$ and $\upsilon^{g}$ represent the measurement noise of the accelerometer and gyroscope, respectively.

### 3.2. IMU Calibration Data Collection and Preprocessing

Accurate division of IMU data into stationary and motion intervals can improve the calibration accuracy of the IMU. To address this issue, this paper exploits the fact that the measured angular velocity of the gyroscope should be close to 0 when the IMU is not moving. It uses a sliding variance test of the angular velocity magnitude to determine the intervals of the collected IMU data. The method sets a window period and moves it back continuously, using the gyroscope output data magnitude as input and using a sliding variance detector to calculate the variance of the data with the mean of that particular interval. The data variance will be below the predefined threshold if the gyro remains stationary. Conversely, the data variance will exceed the threshold if the gyro is moving. By setting an appropriate threshold, unexpected disturbances that could affect the identification of the stationary interval can be effectively minimized. Given a gyroscope output data sequence $\omega \left(t_{1}\right),\omega \left(t_{2}\right)\cdots \omega \left(t_{n}\right)$ and a sampling window size m, the variance constraint at time $t_{k}$ for determining the gyroscope as being in a stationary state is as follows:

(8) $$\delta^{2}=\frac{1}{2m+1}\sum_{\;}^{k+m}{\left(\|\omega \left(t_{k}\right)\|-E\left(\omega \left(t_{k}\right)\right)\right)}^{2}\leq G$$

here $\delta^{2}$ represents the sliding variance of the angular velocity magnitude, $E\left(\omega \left(t_{k}\right)\right)$ is the mean magnitude of the data within the sliding window at the time $t_{k}$, and G is the threshold value set.

When collecting IMU calibration data, it is important to ensure that the IMU remains stationary for at least one minute. This allows sufficient static gyro data to be collected to determine the appropriate threshold. The IMU array should be rotated to different orientations to collect a comprehensive data set. Each rotation should take approximately 2 s; after each rotation, a stationary state should be maintained for approximately 30 s. This entire process should be repeated for a total of 89 rotations.

**Figure 4:**
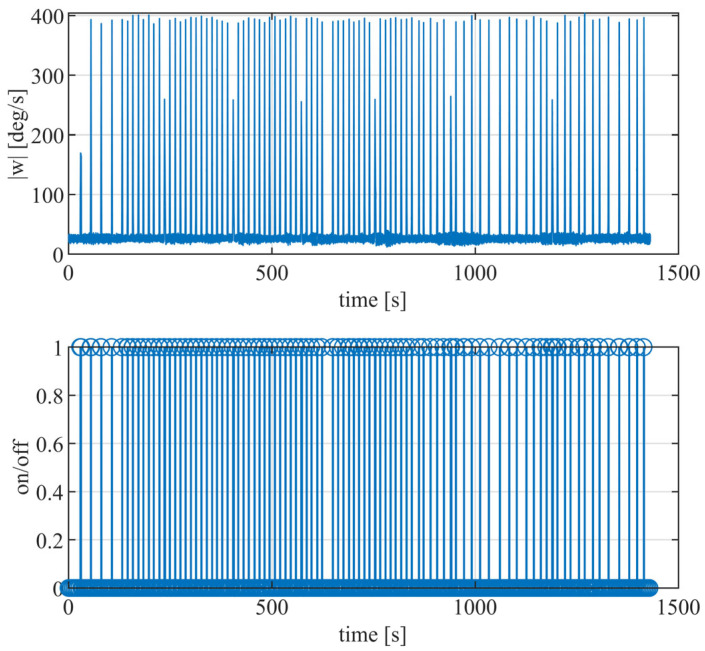
The **upper** figure shows the angular velocity magnitude for static interval detection, and the **lower** figure shows the division of stationary and motion intervals.

The collected data are pre-processed and segmented into stationary and moving intervals. Figure 4 illustrates this process, with the upper part representing the magnitude of the angular velocity. In contrast, the lower part shows the corresponding stationary and motion intervals, with empty intervals representing stationary periods and blue intervals representing motion periods. A total of 89 rotations were carried out, resulting in 89 motion intervals that correspond to the actual motion situations. This paper applied fine-tuning to the end points of the fixed intervals to ensure the accuracy of the segmentation points between the stationary and motion intervals. The accuracy of the segmentation points was determined based on the calibration results of the accelerometer. Multiple experimental validations confirmed that the accelerometer calibration achieved higher accuracy at the average segmentation points. These results indicate that by analyzing the angular velocity magnitude, the stationary and motion intervals can be accurately distinguished without missed detections, thereby ensuring the accuracy of the IMU array calibration.

### 3.3. Estimation of IMU Calibration Parameters

After segmenting the calibration data into stationary and motion intervals, it is necessary to estimate the systematic error parameters for the accelerometer and gyro separately. For the calibration of the accelerometer in the IMU, the unknown parameter vector to be estimated is as follows:

(9) $$x^{f}={\left[\begin{array}{ccccccccc} \alpha_{yz} & \alpha_{zy} & \alpha_{yz} & s_{x}^{f} & s_{y}^{f} & s_{z}^{f} & b_{x}^{f} & b_{y}^{f} & b_{z}^{f} \end{array}\right]}^{T}$$

During the calibration process, the mean of each stationary interval is taken to ignore the noise. Let the Equation (6) represent this process:

(10) $$f^{B}=h\left(f^{b_{i}},x^{f}\right)=T^{f}K^{f}\left(f^{b_{i}}+b^{f}\right)$$

The static interval data from M positions of the IMU array are averaged with the stationary interval data at each position. The difference between the averaged data and the local gravity acceleration is then used to estimate the parameters that minimize the error. The cost function for this estimation is as follows:

(11) $$L\left(x^{f}\right)={\sum_{k=1}^{M}\left({\left\|g\right\|}^{2}-{\left\|h(f_{k}^{b_{i}},x^{f})\right\|}^{2}\right)}^{2}$$

The parameter values can be estimated by minimizing the cost function using the Levenberg–Marquardt (L.M.) algorithm.

Retain the gyroscope data from the IMU array while it is powered on and stationary, and take an average to obtain the gyroscope bias parameters. The vector of other unknown parameters is as follows:

(12) $$x^{g}={\left[\begin{array}{ccccccccc} \gamma_{yz} & \gamma_{zy} & \gamma_{xz} & \gamma_{zx} & \gamma_{xy} & \gamma_{yx} & s_{x}^{g} & s_{y}^{g} & s_{z}^{g} \end{array}\right]}^{T}$$

By observing the changes in the direction of gravity between two successive stationary intervals, it is possible to determine the angle of rotation of the gyroscope during the interval of movement. The parameters that minimize the difference between this derived angle and the angle obtained by integrating the angular velocity can be estimated as follows:

(13) $$u_{g,k}=Q\left[\omega_{i}^{b_{i}},u_{f,k-1}\right]$$

here $u_{f,k-1}$ represents the vector of gravity direction on each axis of the previous stationary interval, provided by the calibrated accelerometer. $u_{g,k}$ represents the vector of gravity direction on each axis of the current stationary interval, Q represents the fourth-order Runge–Kutta integration algorithm [38], based on the gravity direction vector on each axis of the previous stationary interval, to integrate the measurement values of the gyroscope and obtain the gravity direction vector on each axis at the current moment. This research quaternions to represent rotations, and the quaternion kinematic differential equation is as follows:

(14) $$h\left(q,t\right)=\dot{q}=\frac{1}{2}\Omega \left(\omega \left(t\right)\right)q$$

where $\Omega \left(\omega \right)$ represents the skew-symmetric matrix of angular velocity, that is:

(15) $$\Omega \left(\omega \right)=\left[\begin{array}{cccc} 0 & -\omega_{x} & -\omega_{y} & -\omega_{z}\\ \omega_{x} & 0 & \omega_{z} & -\omega_{y}\\ \omega_{y} & -\omega_{z} & 0 & \omega_{x}\\ \omega_{z} & \omega_{y} & -\omega_{x} & 0 \end{array}\right]$$

The fourth-order Runge–Kutta integration algorithm is as follows:

(16) $$q_{k+1}=q_{k}+\;t\frac{1}{6}\left(k_{1}+2k_{2}+2k_{3}+k_{4}\right)$$

(17) $$k_{i}=h\left(q^{\left(i\right)},t_{k}+c_{i}\;t\right)$$

(18) $$\left\{\begin{array}{l} q^{\left(i\right)}=q_{k}\;\left(if\;i=1\right)\\ q^{\left(i\right)}=q_{k}+\;t\sum_{j=1}^{i-1}a_{ij}k_{j}\;(if\;i>1) \end{array}\right.$$

where $c_{i},a_{ij}$ is as follows:

$$\begin{array}{l} c_{1}=0,c_{2}=\frac{1}{2},c_{3}=\frac{1}{2},c_{4}=1,\\ a_{21}=\frac{1}{2},a_{31}=0,a_{41}=0,\\ a_{32}=\frac{1}{2},a_{42}=0,a_{43}=1 \end{array}$$

At last, after each computation, the quaternions need to be normalized, and the cost function is as follows:

(19) $$L\left(x^{g}\right)=\sum_{k=2}^{M}\|u_{f,k}-u_{g,k}{\|}^{2}$$

Similarly, the parameters of interest can be estimated by minimizing the cost function using the Levenberg–Marquardt algorithm.

By substituting the estimated parameter values from the accelerometer and gyroscope into Equations (6) and (7), the calibrated IMU measurements can be obtained. To validate the calibration method proposed in this paper, subsequent evaluations are carried out to assess its static and dynamic performance.

## 4. Data Fusion Estimation and Error Analysis in Pedestrian Navigation Systems

### 4.1. Data Fusion Estimation in Pedestrian Navigation Systems

**Figure 5:**
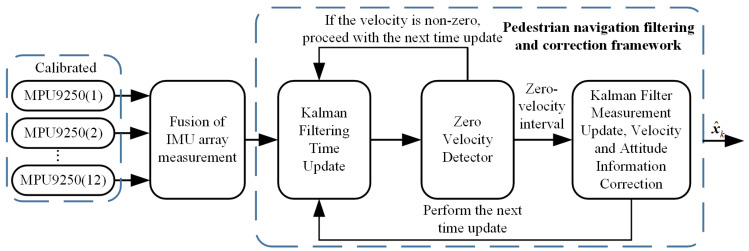
Filtering and Correction Framework of Pedestrian Navigation System Based on IMU Array.

The calibrated and compensated IMU data are fused, and the pedestrian state information is estimated under the correction of the ZUPT algorithm. As shown in Figure 5, after the fusion of the measurements from the IMU array, the data enter the pedestrian navigation filtering correction framework, which consists of Kalman filtering and the ZUPT algorithm. First, the next state value is predicted based on the system transition matrix. Second, the predicted velocity is judged using a zero-velocity detector. If it is a non-zero-velocity interval, the time update for the next moment is performed; otherwise, the measurement update is performed to correct the predicted velocity and other state information. Finally, the estimated state information of the pedestrian’s motion is obtained.

In this paper, the observation domain fusion method is used to fuse the measurement values of the IMU array to compensate for random errors and thereby improve the accuracy of pedestrian navigation. First, the measurement values from different IMU coordinate systems $b_{i}$ on the IMU array are transformed into the array coordinate system B. This can compensate for errors caused by the different distribution positions of the accelerometers on the array. Second, the calibrated measurement values from each IMU on the array in the same coordinate system are averaged, and the fused data are used for the pedestrian navigation system. Although we adopt the averaging fusion method to fuse the measurement values of the IMU array and can only achieve suboptimal performance, it has lower computational complexity than the least squares method and is easier to implement in engineering.

ZUPT is an important correction method in pedestrian navigation systems, consisting of a zero-speed detector and zero-speed update. The zero-velocity detector identifies the state of pedestrian footsteps based on sensor data and distinguishes between stationary and moving footsteps. The ZUPT uses the detection results of the zero-velocity detector to update the velocity information of the inertial measurement unit (IMU) to eliminate error accumulation and improve localization accuracy. The accuracy of the zero-velocity detector is critical to the effectiveness of the entire algorithm. Commonly used detectors include acceleration magnitude detection, sliding variance, and angular velocity detection, each of which can have false negatives due to single-source information. Generalized likelihood detection combines sliding variance, angular velocity detection, and acceleration magnitude detection, resulting in more accurate and reliable detection results [39].

(20) $$T_{k}=\frac{1}{W}\sum \left(\frac{1}{{\left\|\sigma_{a}\right\|}^{2}}{\left\|y^{a}-g\frac{{\bar{y}}^{a}}{\left\|{\bar{y}}^{a}\right\|}\right\|}^{2}+\frac{1}{\left\|\sigma_{g}^{2}\right\|}{\left\|y_{g}\right\|}^{2}\right)$$

where W is the window length of the data, $\sigma_{a},\sigma_{g}$ are the measurement errors of the accelerometer and gyroscope, respectively, and ${\bar{y}}_{a},{\bar{y}}_{g}$ are the average values of acceleration and angular velocity within the window.

ZUPT combined with Kalman filtering forms the ZUPT–Kalman filter. The state-space estimation model of this paper is as follows:

(21) $$x={\left[\begin{array}{ccc} p & v & \theta \end{array}\right]}^{T}$$

where $p={\left[\begin{array}{ccc} p_{x} & p_{y} & p_{z} \end{array}\right]}^{T}$ represents the position information of the three axes, $v={\left[\begin{array}{ccc} v_{x} & v_{y} & v_{z} \end{array}\right]}^{T}$ represents the velocity information of the three axes, and $\theta ={\left[\begin{array}{ccc} \theta_{x} & \theta_{y} & \theta_{z} \end{array}\right]}^{T}$ represents the angles of rotation along the three axes.

The discrete model of the ZUPT-Kalman filter at a time k is as follows:

(22) $$\left\{\begin{array}{l} x_{k}=F_{k-1}x_{k-1}+G_{k-1}w_{k-1}\\ z_{k}=H_{k}x_{k}+n_{k} \end{array}\right.$$

where the Gaussian white noise w,n represents the system noise and measurement noise, respectively. F,G,H,R denote the state transition matrix, noise gain matrix, observation matrix, and measurement noise matrix, respectively.

The measurement noise matrix is positive definite.

(23) $$F=\left[\begin{array}{ccc} 0_{3\times 3} & I_{3\times 3} & 0_{3\times 3}\\ 0_{3\times 3} & 0_{3\times 3} & f_{n}\\ 0_{3\times 3} & 0_{3\times 3} & 0_{3\times 3} \end{array}\right]$$

(24) $$G=\left[\begin{array}{ll} 0_{3\times 3} & 0_{3\times 3}\\ C_{b}^{n} & 0_{3\times 3}\\ 0_{3\times 3} & -C_{b}^{n} \end{array}\right]$$

(25) $$H=\left[\begin{array}{ccc} 0_{3\times 3} & I_{3\times 3} & 0_{3\times 3} \end{array}\right]$$

here $f_{n}=f_{b}C_{b}^{n}$ represents the transformation of the measured acceleration from the vehicle coordinate system to the navigation coordinate system and $C_{b}^{n}$ represents the rotation matrix from the vehicle coordinate system to the navigation coordinate system.

By applying the Kalman filter model in this paper to the ZUPT–Kalman filter algorithm for state estimation, the final navigation information can be obtained. The system state estimation process at a time k is as follows. First, the time update for the state prediction equation is as follows:

(26) $${\hat{x}}_{k}=F_{k-1}{\hat{x}}_{k-1}+G_{k-1}w_{k-1}$$

where ${\hat{x}}_{k},{\hat{x}}_{k-1}$ represent the estimated state quantities at times k and k − 1, respectively.

Next, the time update for the error covariance matrix P is as follows:

(27) $$P_{k|k-1}=F_{k,k-1}P_{k-1}F_{k,k-1}^{T}+G_{k-1}Q_{k-1}G_{k-1}$$

First, calculate the Kalman filter gain $K_{k}$ as:

(28) $$K_{k}=\frac{P_{k|k-1}H_{k}^{T}}{H_{k}P_{k|k-1}H_{k}^{T}+R_{k}}$$

The measurement update for the state vector and error covariance matrix is as follows:

(29) $${\hat{x}}_{k}={\hat{x}}_{k|k-1}+K_{k}\left(z_{k}-H_{k}{\hat{x}}_{k|k-1}\right)$$

(30) $$P_{k}=\left(I_{9\times 9}-K_{k}H_{k}\right)P_{k|k-1}$$

Upon obtaining the estimated state vector ${\hat{x}}_{k}$ at time k, a Kalman filtering process within a zero-velocity interval is completed. Iterations continue iteratively to obtain final navigation information, such as the pedestrian’s ultimate position during walking.

### 4.2. Error Analysis in Pedestrian Navigation Systems

The errors in pedestrian navigation systems primarily stem from measurement errors in sensors and errors in ZUPT algorithms. Therefore, this paper will analyze the noise performance of sensors and the sources of errors in ZUPT algorithms separately. After fusing the measurement values from multiple IMUs, the noise performance of the array can be quantitatively analyzed. All IMUs on the array are of the same model, and theoretically, each IMU exhibits equivalent noise performance. Therefore, the calculation of measurement noise of the IMU array is derived as follows:

(31) $$w_{A}=\frac{1}{N}\sum_{i=1}^{N}w_{i},\sigma_{i}^{2}=E\left[{\left(w_{i}\right)}^{2}\right]$$

(32) $$\sigma_{A}^{2}=E\left[{\left(\frac{1}{N}\sum_{i=1}^{N}w_{i}\right)}^{2}\right]=\frac{1}{N^{2}}E\left[\sum_{i=1}^{N}{\left(w_{i}\right)}^{2}\right]=\frac{1}{N}\sigma_{i}^{2}$$

where $w_{A}$ represents the measurement noise of the array with a variance of $\sigma_{A}$ and $w_{i}$ represents the measurement noise of the ith IMU in the array with a variance of $\sigma_{i}$. From Equation (32), it can be derived that the standard deviation of the measurement noise of the IMU array is $1/\sqrt{N}$ times the standard deviation of an individual IMU, indicating that a factor improves the performance of the IMU array $\sqrt{12}$ compared to a single IMU.

The instability of IMU bias, angular velocity, and velocity random walk are crucial indicators for evaluating the random noise performance of an IMU. This paper uses the Allan variance method to quantitatively analyze the IMU accelerometer measurements. The Allan variance is a widely adopted technique for quantifying random errors in IMUs. Initially, the static data generated by the IMU is divided into segments with a specific interval. Subsequently, the average value within each interval is computed, and the differences between adjacent segments are determined. Ultimately, the random errors of the static data can be depicted in a graph, enabling simple calculations to derive the parameters of the IMU’s random errors.

**Figure 6:**
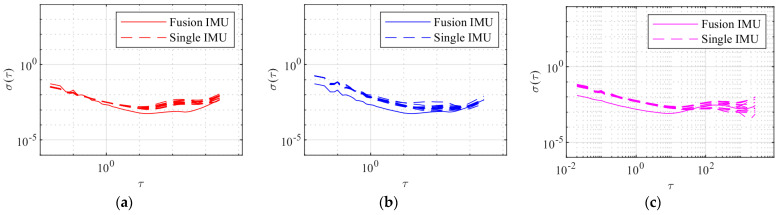
Accelerometer Allan variance analysis. (**a**) Accelerometer x-axis, (**b**) accelerometer y-axis, and (**c**) accelerometer z-axis.

Place the IMU array on a stable horizontal surface and ensure data are collected in a static environment with a consistent indoor temperature. Continuously collect static data for 4 h. Analyze the collected data using Allan variance analysis, as demonstrated in Figure 6. Utilize the Allan variance method to analyze the accelerometer measurements along all three axes. The Allan variance is a function of the period $\tau =m\tau_{0}$, calculated for different values τ to determine the corresponding Allan variance. The values $\log_{10}\left(\tau \right)$ are taken as the x-axis and $\log_{10}\left(\sigma \left(\tau \right)\right)$ as the y-axis. The solid line on the graph represents the fused Allan variance, while the dashed line represents the Allan variance of a single IMU. The point at which the slope of the double logarithmic curve in the Allan variance plot reaches 0 indicates the minimum value position and identifies the in-run instability.

According to the analysis of the Allan variance plot in Table 2, the in-run instability of the accelerometer’s x-axis fused measurement values improved by 3.04 times compared to the average accuracy of the in-run instability of the 12 IMUs. The in-run instability of the accelerometer’s y-axis fused measurement values improved by 3.07 times compared to the average accuracy of the in-run instability of the 12 IMUs. The in-run instability of the accelerometer’s z-axis fused measurement values improved by 2.92 times compared to the average accuracy of the in-run instability of the 12 IMUs. The average triaxial noise performance improvement factor indicates that the fused accelerometer exhibits a 3.01-fold increase in noise performance compared to a single IMU. The experimental results indicate that after fusing the data from the three axes, the variability of the bias is nearly $1/\sqrt{12}$ times lower than that of a single IMU, resulting in a significant enhancement of compensation for random error suppression.

**Figure 7:**
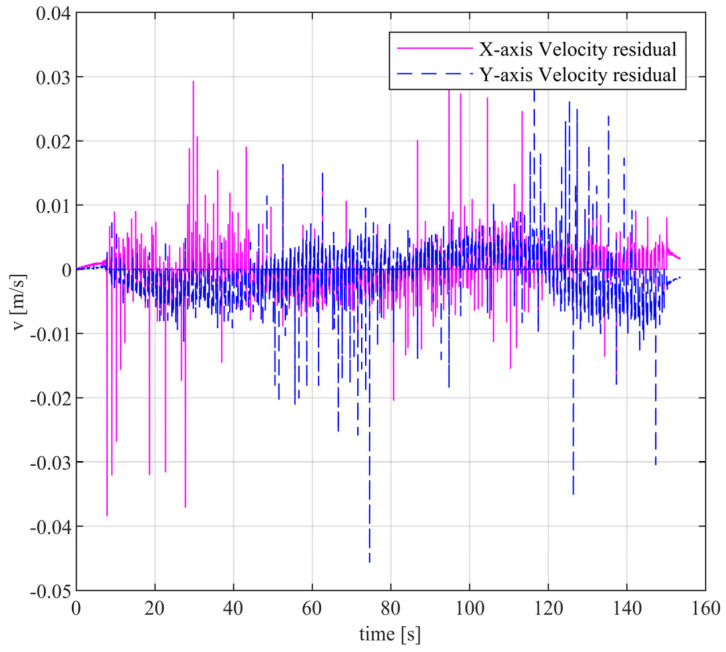
After ZUPT, the velocity residuals are depicted, with blue indicating the velocity residual on the x-axis and a purple dashed line indicating the velocity residual on the y-axis.

ZUPT primarily adjusts the velocity and attitude. However, in practical testing, even after ZUPT, the residual velocity in the zero-velocity interval is not zero, resulting in inadequate correction of position errors. Figure 7 shows the velocity residual of the zero-velocity interval after ZUPT when walking around a closed interval. By statistically analyzing the velocity residuals in the same walking direction, we can obtain the average value of velocity residuals in the same walking direction as $7.5\times 10^{-3}\;m/s$. Although the residual is small, it can accumulate over time and result in significant errors. For example, walking for 100 s will produce an error of 0.75 m. In this paper, a simple and effective method is adopted to correct the position error caused by velocity residuals. This method involves treating the displacement increment as zero when the feet of the rigidly connected IMU array are in the zero-velocity interval, serving as a pseudo-measurement to correct the position error caused by velocity residuals.

## 5. Pedestrian Navigation Experiment and Analysis

The improved performance of pedestrian navigation after error compensation was evaluated in this paper using two walking trajectories. The first trajectory consisted of a straight path over a distance of 80 m, while the second trajectory was a closed-loop path of approximately 140 m. A custom-designed IMU array was attached to the front of the shoes to collect and store data from the accelerometer and gyroscope outputs. This array was connected to a laptop computer via a USB, allowing for data collection at a rate of 50 Hz. To fully capture the pedestrian’s motion information, the walking speed of the test subjects was slower compared to normal walking speed. Offline data analysis was then performed using a combination of Kalman filtering and ZUPT algorithms to estimate the pedestrian’s position and orientation information.

### 5.1. Straight-Line Walking Experiment

**Figure 8:**
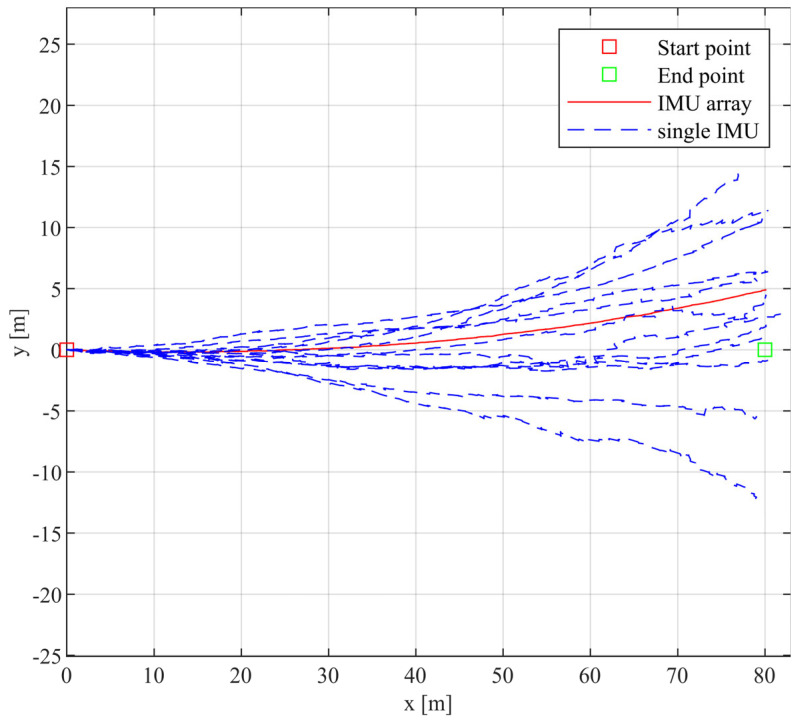
In linear walking trajectory, the solid red line represents the trajectory of the IMU array, and the dashed blue line represents the trajectory of a single IMU.

The straight-walking experiment data were collected along the predetermined 80-m trajectory. After IMU calibration, Figure 8 illustrates the experimental trajectory of straight walking, with the x-axis representing the reference trajectory ranging from 0 m to 80 m. The horizontal error between the estimated trajectory and the reference trajectory end point’s position was calculated to evaluate the straight-walking experiment.

After analyzing Figure 8, we obtained Table 3. At distances of 40 m and 80 m, we performed a quantitative analysis of the horizontal errors between the estimated trajectories of each IMU and the reference trajectory for both individual IMUs and the entire array. At the 40 m position, the array’s horizontal error is 0.53 m, which is 69.19% lower than the average horizontal error of all IMUs (1.72 m). At the 80 m position, the array’s horizontal error is 4.88 m, 25.57% lower than the average IMU error (6.56 m). This indicates that as the walking distance increases, the impact of IMUs with more significant measurement errors on the navigation accuracy of the array gradually increases. The straight walking trajectory is symmetrically distributed relative to the reference trajectory, suggesting that the IMU calibration method proposed in this paper effectively compensates for the systematic errors of each IMU. However, despite calibration compensating for systematic errors, random drift errors still occur during the walking process, leading to discrepancies between the estimated walking trajectory and the reference trajectory. The maximum error in the vertical direction of the reference trajectory is 14.75 m.

### 5.2. Closed-Loop Walking Experiment

The designed closed-loop track has a total length of approximately 140 m and encompasses the perimeter of the experimental building for conducting the walking test. Unlike the straight-line walking experiments, the closed-loop walking experiments do not have a reference track. Therefore, the evaluation is based on the horizontal distance between the start and end points.

Calibrating and fusing the measurement data from each IMU in the IMU array can improve pedestrian navigation performance. To assess the impact of this improvement, this paper fuses measurement data from IMUs before and after calibration. We conducted closed-loop walking experiments to quantitatively evaluate the horizontal error between the start and end points. Since the IMU generates different random error characteristics each time it is turned on, this study included 5 closed-loop walking experiments. The horizontal errors of both the single IMU and the IMU array before and after calibration and data fusion are summarized in Table 4.

Based on the findings presented in Table 4, the IMU array has a significantly reduced horizontal position error compared to a single IMU. Before calibration compensation, the average horizontal position error of a single IMU is 6.25 m, which is reduced to 4.53 m after calibration. The calibration compensation system effectively improves the dynamic performance of the single IMU, with a 27.52% reduction in the average horizontal position error of the IMU after calibration. Similarly, the average horizontal position error of the IMU array measurements is 3.07 m before calibration and 1.52 m after calibration, indicating a lower post-calibration position error compared to the pre-calibration measurement. Regardless of whether the IMU system error is calibrated and compensated, the array consistently shows a lower horizontal position error compared to a single IMU. However, prior to calibration, the average horizontal position error of the IMU array was only 2.03 times greater than that of a single IMU, failing to reach the theoretical value of $\sqrt{12}$ times. After IMU calibration compensation, the average horizontal error of the IMU array increases to 2.98 times that of a single IMU, approaching the expected theoretical $\sqrt{12}$ times. These results indicate that calibrating and compensating each IMU within the array effectively improves the accuracy of the measurements, thereby improving the performance of pedestrian navigation.

**Figure 9:**
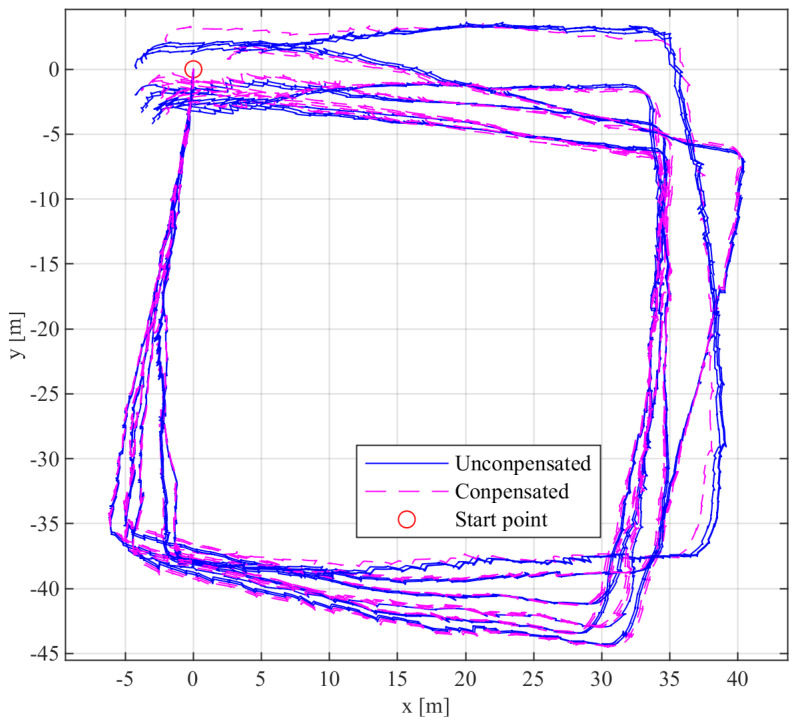
The closed-loop walking experiment trajectory of the single IMU.

The post-calibrated closed-loop walking trajectory of the IMU is depicted in Figure 9, where the blue line represents the walking trajectory without velocity residual compensation, and the purple dashed line represents the walking trajectory with velocity residual compensation.

The analysis of the horizontal position error in the closed-loop walking experiment before and after velocity residual compensation for each IMU in Figure 9 is shown in Table 5. Before compensation, the average horizontal position error for a single IMU is 4.27 m, while after compensation, the average horizontal error for a single IMU is 3.54 m. Compensation for velocity residuals reduces the average horizontal position error by 0.73 m.

**Figure 10:**
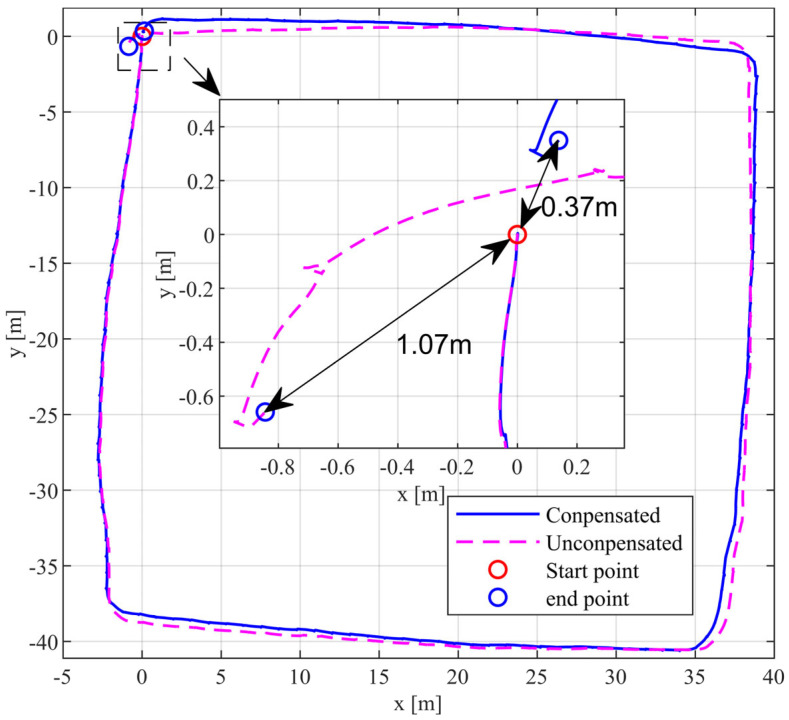
The closed-loop walking experiment trajectory of the IMU array.

As shown in Figure 10, the blue trajectory represents the trajectory after compensation for the velocity residuals generated by the ZUPT. Conversely, the purple trajectory represents the trajectory without velocity residual compensation, while both trajectories have undergone calibration to correct systematic errors. The horizontal position error between the start and end points of the blue trajectory is 0.37 m, while that of the purple trajectory is 1.07 m. By compensating for the velocity residuals, the horizontal position error is reduced by 0.7 m compared to the uncompensated position error. This indicates that compensating for the velocity residuals after ZUPT can improve the accuracy of pedestrian navigation positioning information. Furthermore, the improvement effect becomes more pronounced as the walking time increases. In this approximately 145 m closed-loop walking experiment, the accuracy improved by 65.42% compared to when the ZUPT velocity residuals remained uncompensated.

## 6. Conclusions

This paper focuses on improving the navigation accuracy of a pedestrian navigation system based on a self-developed IMU array system. Starting from the calibration of IMU system errors and the compensation of ZUPT velocity residuals, error compensation is performed to improve navigation accuracy. To calibrate the IMUs, a multi-position independent calibration method is developed. This method uses a sliding variance detector to detect the magnitude of angular velocity, allowing more accurate segmentation of stationary intervals. It improves the accuracy of position calibration without external devices. The velocity residuals after ZUPT are analyzed and the resulting position errors are compensated.

Experimental results indicate a significant improvement in the average noise performance of the calibrated IMU array, which improves by 3.01 times in terms of static noise. In the closed-loop walking experiment, horizontal position errors decrease by 64.52% after compensating for the velocity residual. Compared to individual IMUs, the calibrated IMU array performs 2.98 times better. Moreover, the calibrated array outperforms the un-calibrated IMU array with a 50.49% reduction in horizontal position errors. The experiment also demonstrates that static and dynamic performances align closely with the theoretically expected results. In summary, the error compensation method proposed in this paper has achieved favorable navigation performance on the self-developed IMU array. Therefore, similar error calibration and compensation methods can be adopted by other IMU array systems to achieve improved navigation performance.

In the straight walking experiment, as the walking time increases, the navigation performance of the IMU array may be influenced by IMUs with higher measurement errors within the array. Therefore, it would be valuable in future work to investigate a method that can adaptively adjust the weights of each IMU in the measurement fusion process.

## Figures and Tables

**Table 1:** Comparison of the self-developed IMU array with the performance of other IMUs.

Comparison Items	Self-Developed IMU Array	MTi-2	ADIS16500
gyroscope noise density/$\deg/s/\sqrt{\mathrm{Hz}}$	0.0029	0.003	0.0061
accelerometer noise density/$\mu g/\sqrt{\mathrm{Hz}}$	87	70	90
IMU price/Dollar	110	320	320

**Table 2:** Analysis of three-axis accelerometer in-run instability.

IMU NO.	$\mathrm{Three}\text{-}\mathrm{Axis}\;\mathrm{In}\text{-}\mathrm{Run}\;\mathrm{Instability}\;10^{-3}\;m/s/\sqrt{h}$		
	x-axis	y-axis	z-axis
1	2.58	2.61	2.57
2	2.65	1.97	2.81
3	2.68	2.66	3.38
4	2.57	2.57	2.76
5	2.46	2.34	2.72
6	2.71	1.75	1.99
7	2.59	2.72	2.32
8	2.74	2.86	1.61
9	2.67	2.25	2.25
10	2.69	2.71	1.78
11	2.79	2.75	1.68
12	2.63	2.67	1.83
average	2.65	2.49	2.31
fused measurement values	0.87	0.81	0.79

**Table 3:** The straight-line walking experiment’s horizontal error between the estimated and reference trajectory end points.

Position/m	End point Horizontal Error/m												
	1	2	3	4	5	6	7	8	9	10	11	12	Array
40	1.02	0.42	1.74	0.03	1.90	4.39	3.48	2.71	1.46	1.58	0.59	1.33	0.53
80	12.16	11.34	5.64	1.92	5.87	5.33	3.33	6.40	2.91	14.75	10.692	2.36	4.88

**Table 4:** The horizontal position error before and after the calibration of the IMU array.

Test	Uncalibrated Single IMU Horizontal Error/m	Calibrated Single IMU Horizontal Error/m	Uncalibrated Array Horizontal Error/m	Calibrated Array Horizontal Error/m
1	6.21	4.21	3.01	1.07
2	6.10	4.53	3.16	1.53
3	6.38	4.27	3.09	1.74
4	6.23	4.97	3.11	1.69
5	6.31	4.71	3.96	1.56
average	6.25	4.53	3.07	1.52

**Table 5:** The analysis of trajectory in closed-loop walking experiments for individual IMUs.

IMU NO.	Horizontal Position Error before Compensation/m	Horizontal Position Error after Compensation/m
1	4.45	3.83
2	4.26	3.57
3	3.65	2.86
4	5.20	4.64
5	4.72	3.96
6	5.06	4.27
7	4.61	3.87
8	4.21	3.37
9	3.77	2.93
10	3.74	3.01
11	3.25	2.62
12	4.26	3.55
average	4.27	3.54

## Data Availability

Data are contained within the article.

## References

[1] El-Sheimy N., Li Y. (2021). Indoor Navigation: State of the Art and Future Trends. Satell. Navig..

[2] Zhang R., Höflinger F., Reindl L. (2013). Inertial Sensor Based Indoor Localization and Monitoring System for Emergency Responders. IEEE Sens. J..

[3] Harle R. (2013). A Survey of Indoor Inertial Positioning Systems for Pedestrians. IEEE Commun. Surv. Tutor..

[4] Fischer C., Gellersen H. (2010). Location and Navigation Support for Emergency Responders: A Survey. IEEE Pervas. Comput..

[5] Ahn H., Ko K.H. (2009). Simple Pedestrian Localization Algorithms Based on Distributed Wireless Sensor Networks. IEEE Trans. Ind. Electron..

[6] Leppäkoski H., Collin J., Takala J. (2013). Pedestrian Navigation Based on Inertial Sensors, Indoor Map, and WLAN Signals. J. Signal Process. Syst..

[7] Feng D., Wang C., He C., Zhuang Y., Xia X. (2020). Kalman-Filter-Based Integration of IMU and UWB for High-Accuracy Indoor Positioning and Navigation. IEEE Internet Things J..

[8] Kunhoth J., Karkar A., Al-Maadeed S., Al-Ali A. (2020). Indoor Positioning and Wayfinding Systems: A Survey. Hum.-Centr. Comput. Inf. Sci..

[9] Wang X., Zhuang Y., Zhang Z., Cao X., Qin F., Yang X. (2023). Tightly Coupled Integration of Pedestrian Dead Reckoning and Bluetooth Based on Filter and Optimizer. IEEE Internet Things J..

[10] Qiu S., Wang Z., Zhao H., Qin K., Li Z., Hu H. (2018). Inertial/Magnetic Sensors Based Pedestrian Dead Reckoning by Means of Multi-sensor Fusion. Inform. Fusion.

[11] Tang C., Wang Y., Zhang L., Zhang Y., Song H. (2022). Multisource Fusion UAV Cluster Cooperative Positioning Using Information Geometry. Remote Sens..

[12] Tang C., Wang C., Zhang L., Zhang Y., Song H. (2022). Multivehicle 3D Cooperative Positioning Algorithm Based on Information Geometric Probability Fusion of GNSS/Wireless Station Navigation. Remote Sens..

[13] Winter S., Tomko M., Vasardani M., Richter K., Khoshelham K., Kalantari M. (2019). Infrastructure-Independent Indoor Localization and Navigation. ACM Comput. Surv..

[14] El-Sheimy N., Youssef A. (2020). Inertial Sensors Technologies for Navigation Applications: State of the Art and Future Trends. Satell. Navig..

[15] Xie D., Jiang J., Yan P., Wu J., Li Y., Yu Z. (2023). A Novel Three-Dimensional Positioning Method for Foot-Mounted Pedestrian Navigation System Using Low-Cost Inertial Sensor. Electronics.

[16] Wang Y., Chernyshoff A., Shkel A.M. (2020). Study on Estimation Errors in ZUPT-Aided Pedestrian Inertial Navigation Due to IMU Noises. IEEE Trans. Aerosp. Electron. Syst..

[17] Wang L., Tang H., Zhang T., Chen Q., Shi J., Niu X. (2021). Improving the Navigation Performance of the MEMS IMU Array by Precise Calibration. IEEE Sens. J..

[18] Nilsson J., Skog I. Inertial Sensor Arrays—A Literature Review. Proceedings of the 2016 European Navigation Conference (ENC).

[19] Skog I., Nilsson J., Händel P. An Open-Source Multi Inertial Measurement Unit (MIMU) Platform. Proceedings of the 2014 International Symposium on Inertial Sensors and Systems (INERTIAL).

[20] Xue L., Yang B., Yang X., Yuan D., Wang X., Chang H. (2021). A Redundant Fused MIMU Attitude System Algorithm Based on Two-Stage Data Fusion of MEMS Gyro Clusters Array. Measurement.

[21] Nilsson J., Skog I., Händel P. (2014). Aligning the Forces—Eliminating the Misalignments in IMU Arrays. IEEE Trans. Instrum. Meas..

[22] Blocher L., Mayer W., Arena M., Radović D., Hiller T., Gerlach J., Bringmann O. Purely Inertial Navigation with a Low-Cost MEMS Sensor Array. Proceedings of the 2021 IEEE International Symposium on Inertial Sensors and Systems (INERTIAL).

[23] Song T., Li K., Wu Q., Li Q., Xue Q. (2020). An Improved Self-Calibration Method with Consideration of Inner Lever-Arm Effects for a Dual-Axis Rotational Inertial Navigation System. Meas. Sci. Technol..

[24] Jlailaty H.A., Celik A., Mansour M.M., Eltawil A.M. (2023). IMU Hand Calibration for Low-Cost MEMS Inertial Sensors. IEEE Trans. Instrum. Meas..

[25] Carlsson H., Skog I., Jaldén J. (2021). Self-Calibration of Inertial Sensor Arrays. IEEE Sens. J..

[26] Skog I., Nilsson J., Händel P., Nehorai A. (2016). Inertial Sensor Arrays, Maximum Likelihood, and Cramér–Rao Bound. IEEE Trans. Signal Process..

[27] Qiang S., Jieyu L., Qian Z., Qi W. (2018). RCC-OBE Estimation Fusion Approach for MEMS Gyro Array. J. Beijing Univ. Aeronaut. Astronaut..

[28] Nemec D., Andel J., Simak V., Hrbcek J. (2023). Homogeneous Sensor Fusion Optimization for Low-Cost Inertial Sensors. Sensors.

[29] Wang Y., Lin Y., Askari S., Jao C., Shkel A.M. Compensation of Systematic Errors in ZUPT-Aided Pedestrian Inertial Navigation. Proceedings of the 2020 IEEE/ION Position, Location and Navigation Symposium (PLANS).

[30] Wei W., Gao S., Zhong Y., Gu C., Subic A. (2016). Random weighting estimation for systematic error of observation model in dynamic vehicle navigation. Int. J. Control Autom. Syst..

[31] Zhong Y., Gao S., Wei W., Gu C., Subic A. (2015). Random weighting estimation of kinematic model error for dynamic navigation. IEEE Trans. Aerosp. Electron. Syst..

[32] Gao S., Zhong Y., Wei W., Gu C. (2014). Windowing-based random weighting fitting of systematic model errors for dynamic vehicle navigation. Inform. Sci..

[33] Gao Z., Mu D., Gao S., Zhong Y., Gu C. (2016). Robust adaptive filter allowing systematic model errors for transfer alignment. Aerosp. Sci. Technol..

[34] Zhang X., Zhou C., Chao F., Lin C., Yang L., Shang C. (2022). Low-Cost Inertial Measurement Unit Calibration With Nonlinear Scale Factors. IEEE Trans. Ind. Inform..

[35] Tong Z., Tianjun Z., Renyi H., Jiaqi L. (2023). Online Calibration of RIMU Based on Multistage EKF. IEEE Sens. J..

[36] Qureshi U., Golnaraghi F. (2017). An Algorithm for the In-Field Calibration of a MEMS IMU. IEEE Sens. J..

[37] Tedaldi D., Pretto A., Menegatti E. A Robust and Easy to Implement Method for IMU Calibration without External Equipments. Proceedings of the 2014 IEEE International Conference on Robotics and Automation (ICRA).

[38] Andrle M.S., Crassidis J.L. (2013). Geometric Integration of Quaternions. J. Guid. Control Dyn..

[39] Wahlström J., Skog I. (2021). Fifteen Years of Progress at Zero bias instability Velocity: A Review. IEEE Sens. J..

